# Individuality of the Extremely Premature Infant Gut Microbiota Is Driven by Ecological Drift

**DOI:** 10.1128/msystems.00163-22

**Published:** 2022-04-27

**Authors:** David Seki, Clemens Schauberger, Bela Hausmann, Angelika Berger, Lukas Wisgrill, David Berry

**Affiliations:** a Centre for Microbiology and Environmental Systems Science, Department of Microbiology and Ecosystem Science, Division of Microbial Ecology, University of Vienna, Vienna, Austria; b Department of Pediatrics and Adolescent Medicine, Division of Neonatology, Pediatric Intensive Care and Neuropediatrics, Comprehensive Center for Pediatrics, Medical University of Vienna, Vienna, Austria; c Hadal & Nordcee, Department of Biology, University of Southern Denmark, Odense, Denmark; d Joint Microbiome Facility of the Medical University of Vienna and the University of Vienna, Vienna, Austria; e Department of Laboratory Medicine, Medical University of Vienna, Vienna, Austria; University of Maine

**Keywords:** deterministic, microbiota assembly, premature infants, stochastic

## Abstract

The initial contact between humans and their colonizing gut microbiota after birth is thought to have expansive and long-lasting consequences for physiology and health. Premature infants are at high risk of suffering from lifelong impairments, due in part to aberrant development of gut microbiota that can contribute to early-life infections and inflammation. Despite their importance to health, the ecological assembly and succession processes governing gut microbiome composition in premature infants remained incompletely understood. Here, we quantified these ecological processes in a spatiotemporally resolved 16S rRNA gene amplicon sequencing data set of 60 extremely premature neonates using an established mathematical framework. We found that gut colonization during the first months of life is predominantly stochastic, whereby interindividual diversification of microbiota is driven by ecological drift. Dispersal limitations are initially small but have increasing influence at later stages of succession. Furthermore, we find similar trends in a cohort of 32 healthy term-born infants. These results suggest that the uniqueness of individual gut microbiota of extremely premature infants is largely due to stochastic assembly.

**IMPORTANCE** Our knowledge concerning the initial gut microbiome assembly in human neonates is limited, and scientific progression in this interdisciplinary field is hindered due to the individuality in composition of gut microbiota. Our study addresses the ecological processes that result in the observed individuality of microbes in the gastrointestinal tract between extremely premature and term-born infants. We find that initial assembly is mainly driven by neutral ecological processes. Interestingly, while this progression is predominantly random, limitations to the dispersal of microbiota between infants become increasingly important with age and are concomitant features of gut microbiome stability. This indicates that while we cannot predict gut microbiota assembly due to its random nature, we can expect the establishment of certain ecological features that are highly relevant for neonatal health.

## INTRODUCTION

The human gut microbiota performs a variety of functions essential for the survival of its host. Adults harbor gut microbiota that are generally resistant to perturbation and maintain a stable composition over years and even decades (1). Human-associated microbial lineages are also globally distributed and estimated to have much higher dispersal rates between continents than other terrestrial bacteria (2). However, the human gut microbiota is highly personalized, varying substantially among individuals (3). With birth, the gut microbiota begins assembling *de novo* and eventually stabilizes into a complex community, and it is thought that the initial assembly and succession of the early-life microbiota heavily impact the composition of the resulting stable community (4). However, despite the importance of early-life microorganisms for host health and development, the ecological processes underlying early-life gut microbiota assembly remain poorly understood.

Primary succession begins postdelivery with the arrival of pioneer communities, which shape subsequent steps of succession (5). Over the course of this process, the human host exerts a strong top-down control on colonizing bacteria. Factors such as intestinal transit time (6), diet (7), geographic location (8), host genetics (9), medication use (10), and endocrine (11) and neurological function (12) partially explain interindividual microbiome variation. These factors exert deterministic influence by which species are selected for or expelled from the environment due to differences in their ecological fitness (13). In addition to a variety of deterministic processes driven by the host or interactions within the microbiota (e.g., niche construction), neutral processes can also play a role in shaping ecological systems (14). Stochastic events include the dynamics of passive dispersal (15) and ecological drift (16). For a variety of environments in nonequilibrium states, it is believed that stochasticity dominates initial community assembly, given the general assumption that a broad range of organisms can grow successfully in an uncolonized environment (17).

The gut microbiota of an individual infant can be viewed as a local part of a larger pool of communities found in the infant population, or a so-called metacommunity (18). Local communities differ from each other as a result of species sorting, which can be caused by differences in the combination and magnitude of selection, dispersal, and drift (19). Quantification of the processes that ultimately define the structure and amount of turnover in and between local microbial communities is an important step toward understanding the assembly and succession of the early-life gut microbiota in humans. This understanding is particularly important for the health of premature infants, as aberrant gut microbiota development can contribute to early-life inflammation and adverse outcomes (20). Premature infancy has been rising in incidence worldwide and is one of the leading causes of perinatal morbidity and mortality (21). In premature, as well as term-born, infants, the microbiota has been suggested to develop in a series of successional phases marked by the appearance and disappearance of specific microbial taxa (22, 23), including the eventual establishment of obligate anaerobic bacteria (24), and an early disruptive event such as antibiotic administration can destabilize this process (25). Though some external drivers of microbiota assembly have been identified, we still lack a conceptual framework of assembly processes, and a better understanding of the ecological mechanisms that impose aberrant development of the microbiota is crucial for improving the overall health outcome for premature infants.

In this study, we quantified the ecological processes underlying microbial community assembly in 60 extremely premature infants (born before 28 weeks of gestation and weighing less than 1 kg), based on data obtained in a previously published study (20). We compared our phylogenetic observations to ecological null models to determine the contributions of selection, dispersal, and drift to the observed community turnover (26) in the premature infant gut microbiota. As a comparison, we evaluated a publicly available data set describing the succession of microorganisms in 32 healthy infants born at term age (27). We found that the early-life gut microbiota is initially unstable and low in diversity. However, as succession proceeded, both stability and diversity increased. This progression was driven largely by ecological drift, with a small contribution of deterministic factors and increasing importance of dispersal limitation over time.

## RESULTS

### Indications for deterministic assembly and succession of gut microbiota in extremely premature infants.

Data were obtained from a previously published study of a cohort of 60 extremely premature infants (gestational age, <28 weeks, and birth weight, <1 kg) (20). Stool was sampled from each individual 3, 7, and 14 days postdelivery and then biweekly until discharge from the hospital. We employed 16S rRNA gene-targeted amplicon sequencing and quantitative PCR (qPCR) to characterize microbial composition and quantify total bacterial load, allowing for quantitative microbiome profiling of community composition.

**FIG 1:**
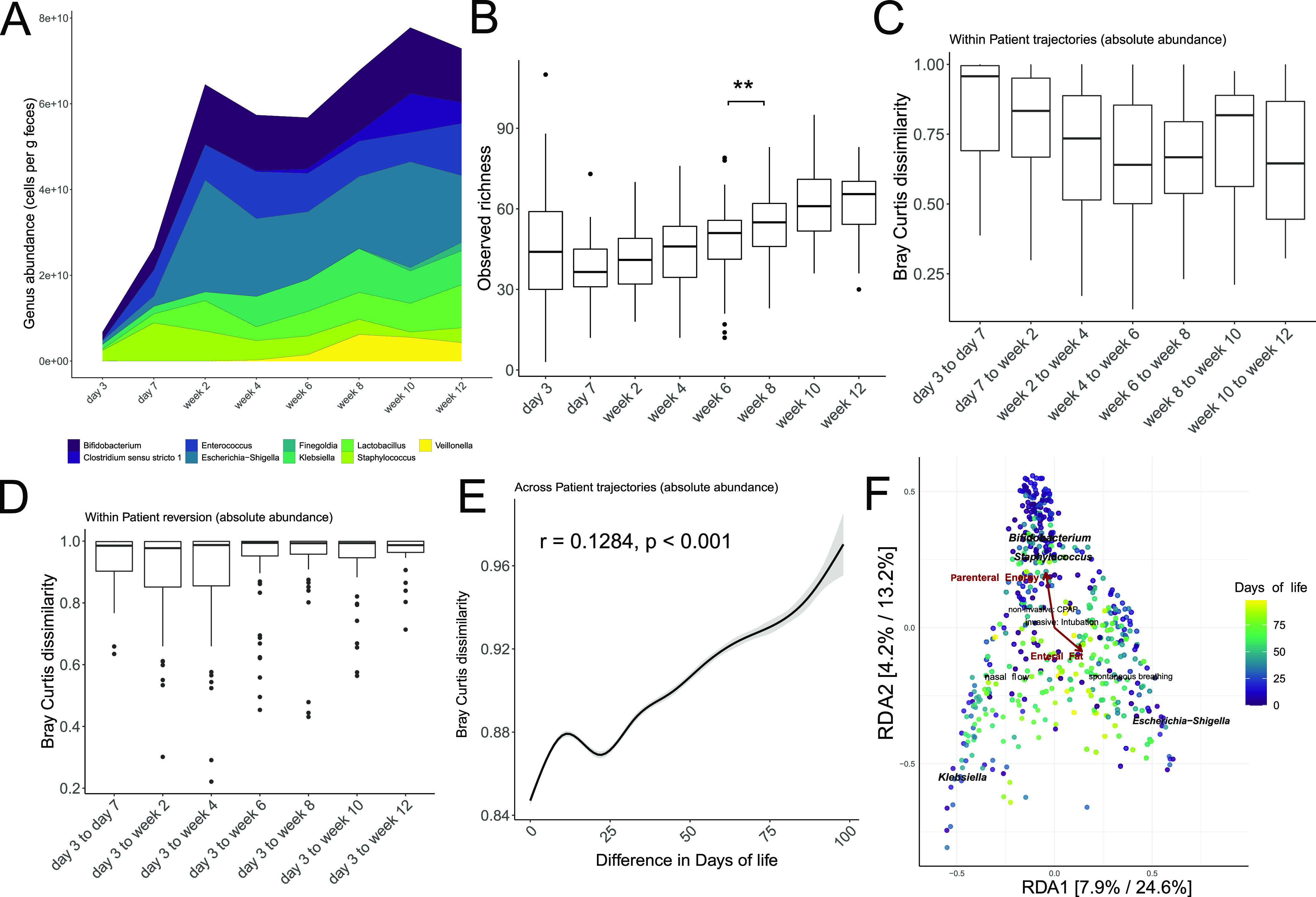
Succession of gut microbiota in extremely premature infants. (A) Absolute abundances of the core genera in premature infants throughout hospitalization. (B) Observed ASV richness. (C) Calculation of Bray-Curtis dissimilarity between adjacent samples of the same individual. (D) Calculation of Bray-Curtis dissimilarity between the first sample and all following samples of the same individual. (E) Mantel correlogram of between-infant Bray-Curtis dissimilarities and the increase of difference in age. (F) Redundancy analysis (RDA) ordination of all 16S rRNA gene amplicon libraries corrected by absolute abundances and 16S rRNA gene copy number. Arrows (red) and text (black) indicate directional contribution of numerical environmental variables that are significantly associated with the observed variation and are positioned with respect to their contribution. Box plots show group median and interquartile range. Smoothed lines result from locally estimated scatterplot smoothing (LOESS) and indicate trends of development. *, *P* < 0.05; **, *P* < 0.01; ***, *P* < 0.001.

We first examined the abundances of the dominant taxa over time, averaged across the patient cohort. Staphylococcus was abundant 3 and 7 days postdelivery but subsequently decreased as total bacterial load plateaued by 2 weeks postdelivery (Wilcoxon, *P* < 0.001; comparison, Staphylococcus abundance at day 7 and week 4) following an expansion of Escherichia*-Shigella* (Wilcoxon, *P* < 0.001; comparison, Escherichia*-Shigella* abundance at day 7 and week 2). Interestingly, by analyzing the relative proportions of amplicon sequence variant (ASV) reads without a correction for total bacterial load, we found that the increase of Staphylococcus during the first week postdelivery was overestimated by the relative abundance data, whereas the subsequent expansion of Escherichia-S*higella* was underestimated compared to the quantitative profile (Fig. S1 in the supplemental material). By 8 to 10 weeks, strictly anaerobic bacteria (*Clostridia*, *Finegoldia*, and *Veillonella*), which were detected only rarely at early time points, increased in abundance (Wilcoxon, *P* = 0.006, 0.07, and 0.0002, respectively; comparison, *Clostridia*, *Finegoldia*, and *Veillonella* abundances at week 6 and week 8) (Fig. 1A). Their occurrence is synchronized to a significant increase of ASV richness between the adjacent time points at week 6 and week 8 (Fig. 1B) (*t* test, *P* = 0.003), providing indications for reproducible, deterministic assembly and succession processes being involved in shaping the early-life microbiota. Furthermore, we observed an overall trend of decreasing Bray-Curtis dissimilarities between samples at adjacent time points from the same individual (Fig. 1C), suggesting that ASV turnover is initially fast and decelerates at later points. Although Bray-Curtis dissimilarities did not significantly differ between adjacent time points, days of life were slightly but significantly associated with the observed variation in microbiota composition (permutational multivariate analysis of variance [PERMANOVA], *R*^2^ = 0.04, *P* = 0.01). Also, we found considerable temporal instability in the microbiota of individual patients, particularly during the first month postdelivery. In some cases, this was due to transient reversions to communities similar to initial (day 3) microbiomes, as indicated by outliers in Fig. 1D. The variation in the microbiome across the cohort tended to increase with age, suggesting that microbiomes were becoming increasingly different from one another (Fig. 1E) (Mantel statistic *r* = 0.1284, *P* < 0.001).

10.1128/msystems.00163-22.1 FIG S1 Relative abundance data for gut microbiota in extremely premature infants. Relative abundances of the core genera in premature infants throughout hospitalization. Download FIG S1, PDF file, 0.05 MB. Copyright © 2022 Seki et al. 2022 Seki et al. https://creativecommons.org/licenses/by/4.0/ This content is distributed under the terms of the Creative Commons Attribution 4.0 International license.

Last, we evaluated whether clinically relevant variables, as summarized in Table 1, could partially explain variations in the microbiota. We first tested a global model for its significance and explanatory power using redundancy analysis (RDA), including all variables listed in Table 1. As the global model was significant (*R*^2^ = 0.53, *P* = 0.001), we proceeded to identify the most significant explanatory variables via stepwise forward selection. Thereby, we identified that patient identity (*P* = 0.01), parenteral energy (*P* = 0.01), mode of ventilation (*P* = 0.01), and enteral fat (*P* = 0.02) partially explain the observed microbiota variation (*R*^2^ = 0.53). We then visualized these results in two-dimensional space by constrained RDA ordination, placing the significant variables relative to their contribution to observed variation within the constrained space. We found that the amount of parenterally delivered dietary energy was associated with levels of *Bifidobacterium* and Staphylococcus. Immediately postdelivery, most extremely premature infants are in need of ventilation support, either invasive (intubation) or noninvasive (continuous positive airway pressure [CPAP]). Spontaneous breathing, a prerequisite to hospital discharge, was associated with Escherichia-*Shigella* levels, while continuous ventilation support via nasal flow was associated with Klebsiella abundances (Fig. 1F).

**TABLE 1:** Subject cohort demographics

Characteristic	Value
No. of individuals	60
% female	63
No. of deceased individuals	7
Birth wt (g)	746.28 ± 153
Body mass index	6.96 ± 0.88
% with connatal infection	50
No. of days of hospitalization	92.85 ± 35.32
Enteral carbohydrates (mg/kg/min)	3.29 ± 1.38
Enteral fats (g/kg/day)	2.69 ± 1.14
Enteral proteins (g/kg/day)	1.47 ± 0.79
Enteral energy (kcal/kg/day)	48.47 ± 20.87
% Spontaneous birth	23
Parenteral carbohydrates (mg/kg/min)	5.14 ± 1.15
Parenteral fats (g/kg/day)	1.46 ± 0.11
Parenteral protein (g/kg/day)	2.59 ± 0.53
Parenteral energy (kcal/kg/day)	54.64 ± 12.97
% intubated	42
Total no. of days of antibiotic intervention	17.08 ± 12
Total dose of antibiotics (mg)	988.413 ± 1,614.15

Altogether, these results provide indications that the gut microbiota composition is highly individual and becomes even more so over time, concomitant with an increase in compositional stability. Interestingly, RDA suggests a bifurcation in the development of microbiota, which is partially attributable to dietary regime as well as ventilation support over the course of hospitalization. Although these variables seem to play a role in the assembly and succession of the premature infant gut microbiota, they still provide only a partial explanation for the observed variation in the microbiota.

### Ecological drift dominates assembly and succession of early-life gut microbiota.

**FIG 2:**
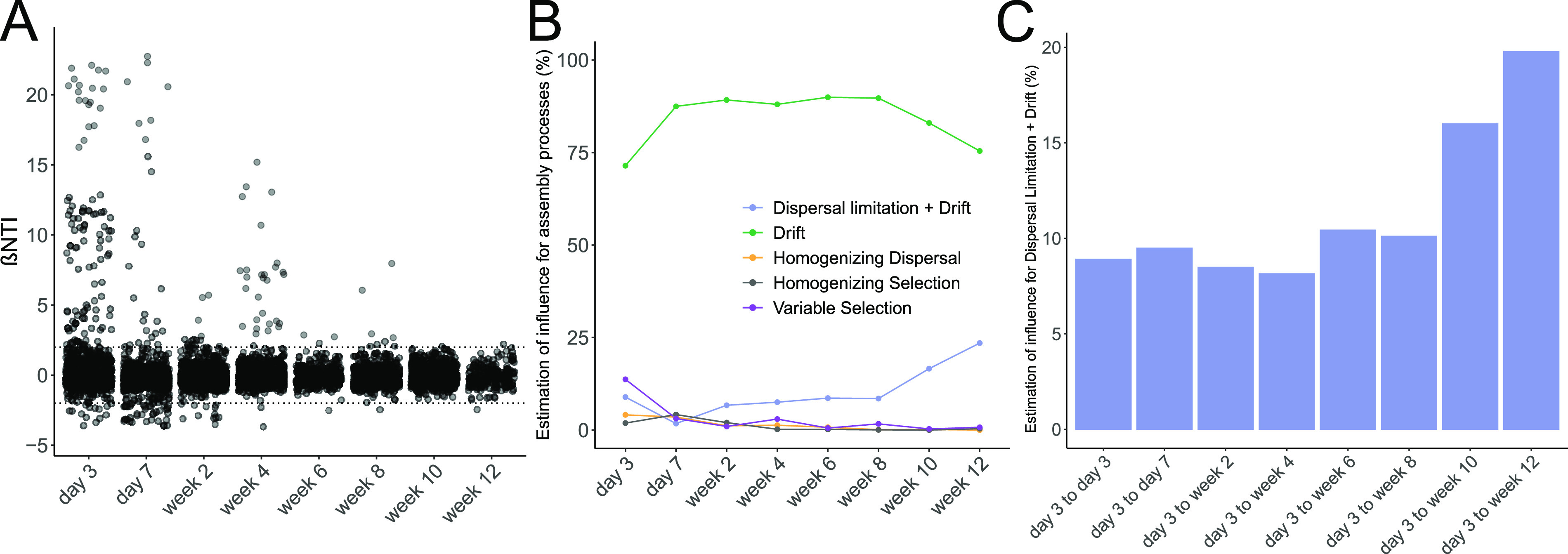
Influence of ecological processes on gut microbiota succession throughout time in extremely premature infants. (A) Distribution of βNTI values for pairwise comparisons of samples between different patients within the same clinical ward. Dashed lines indicate the range of βNTI values under the null hypothesis of no significant effects of selection (<|2|) (B) Contribution of individual turnover processes to observed differences in microbial community composition resulting from pairwise comparisons of samples between different patients sampled during the same calendar week within the same clinical ward. (C) Contribution of dispersal limitation to observed differences in microbial community composition, resulting from pairwise comparisons of samples between different patients who were sampled during the same calendar week between differing clinical wards.

To quantify the contributions of stochastic and deterministic processes in shaping the gut microbiota of this cohort, we calculated the beta mean nearest-taxon index (βNTI) and determined the phylogenetic turnover of microbial communities relative to a null model (26), allowing only for pairwise comparisons of microbiota from different patients who are situated in the same neonatal ward at a given time point. A pairwise comparison of βNTI values indicates that two communities are phylogenetically less similar (greater than 2) or more similar (less than −2) than by random chance and are therefore likely driven by either variable or homogenizing selection. Values greater than |2| are characteristic results of either similar or divergent selective forces that deterministically structure microbiomes. Values equal to or less than |2| indicate stochastic processes as the main forces driving microbial community composition. We observed that stochastic processes are the dominant forces driving species turnover in extremely premature infants. Variable selection, which was present shortly after birth, quickly diminished over the course of hospitalization (Fig. 2A), suggesting that variable selection primarily shapes microbial community structure when gastrointestinal niches are still relatively vacant.

To further analyze the stochastic processes affecting microbiota turnover, we used the modification of Raup-Crick (RC_Bray_) dissimilarity (26). A deviation of RC_Bray_ greater than |0.95| compared to the null model would indicate that communities are assembled by either homogenizing dispersal (RC_Bray_ less than −0.95) or the combination of dispersal limitation and randomly various birth and death rates (drift) (RC_Bray_ greater than 0.95). Homogenizing dispersal and homogenizing selection both appeared to play a minor role throughout most of the hospitalization period. Interestingly, we observed that dispersal limitation gradually increased over time, concomitant with a decrease in drift acting alone (Fig. 2B). This trend was also reflected in the pairwise comparison of all samples to early postdelivery samples, which showed that dispersal limitation between developed and founder microbiota remained below 10% during the first month postdelivery but increased afterward (Fig. 2C). Accordingly, RC_Bray_ values differed significantly between the time point comparisons of day 3 to day 3 and day 3 to week 10 (*t* test, *P* = 0.008).

We conclude that the succession of the extremely premature infant gut microbiota is predominantly stochastic. Species turnover is dominated by drift once the microbiota has reached the carrying capacity of the premature infant gastrointestinal tract (GIT) approximately 2 weeks postdelivery. In addition, an increase in dispersal limitation adds to the effects of drift during later stages of hospitalization. This suggests that stochasticity underlies diversification of the microbiota, whereby exchange of microbes between infants and their environment is pervasive early postdelivery. Only during later stages of hospitalization does dispersal limitation begin to act in concert with drift, thereby impeding the exchange of microbiota between infants. However, the presence of variable selection shortly postdelivery raises the possibility that various priority effects manifest in different infants, a legacy that might translate into diversification during later periods.

### Differences in gestational age at birth underlie early-life variable selection.

**FIG 3:**
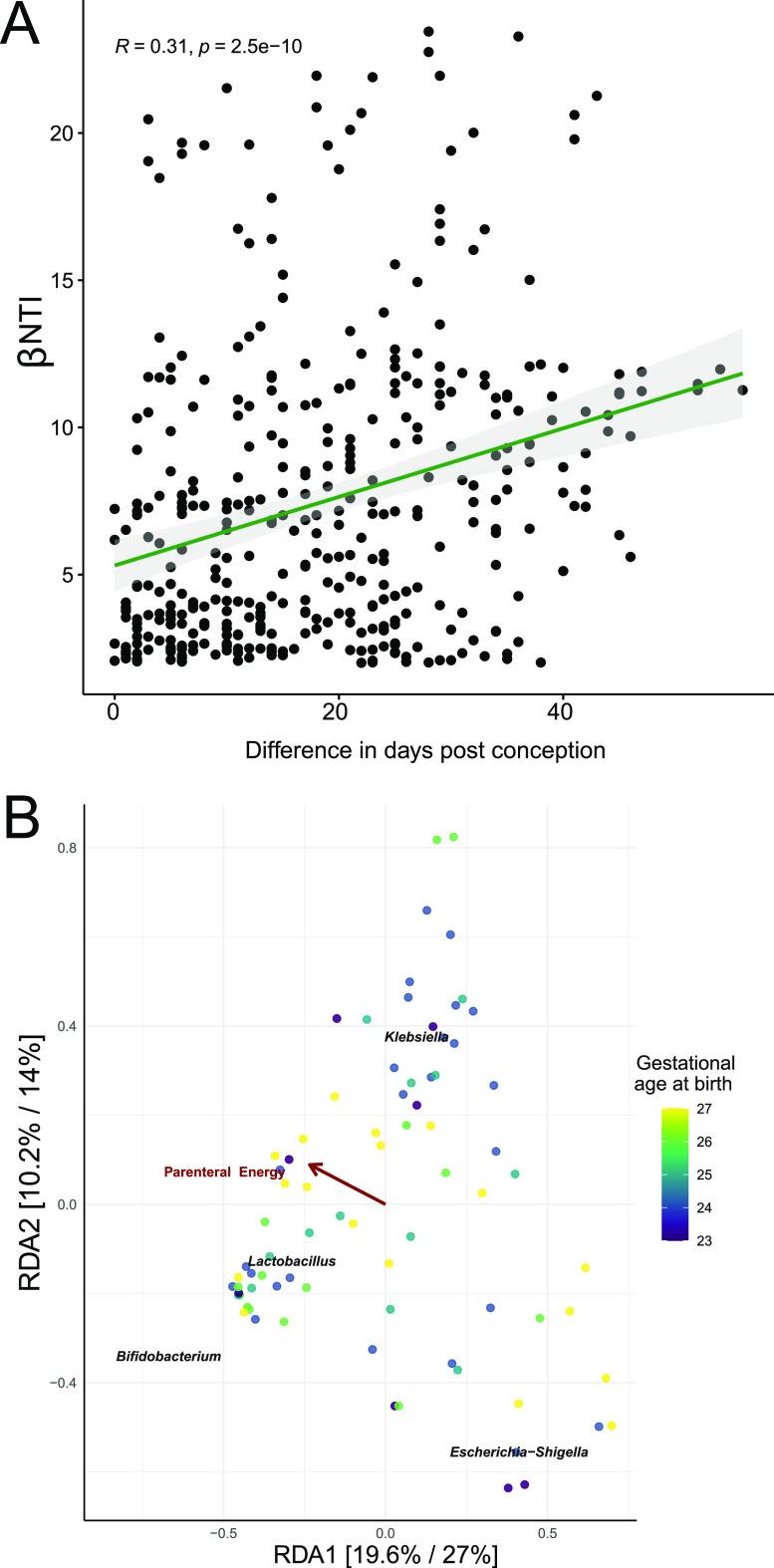
Underlying mechanisms of initial gut microbiota assembly in extremely premature infants. (A) Distribution of βNTI values of >2 for pairwise comparisons of samples between different patients within NICU-1 during the first week postdelivery, regressed against differences in days postconception. Regression line (green) with confidence interval (gray) illustrates the association between βNTI values of >2 and increasing differences between postconceptional age. (B) Redundancy analysis (RDA) ordination of 16S rRNA gene amplicon libraries corrected by absolute abundances and 16S rRNA gene copy number, collected during the first week postdelivery only. Arrows (red) and text (black) indicate directional contribution of numerical environmental variables that are significantly associated with the observed variation and are positioned with respect to their contribution.

Next, we evaluated which factors underlie the observed variable selection immediately postdelivery. βNTI values of >2 were extracted out of all pairwise community comparisons from samples obtained during the first 3 days of life. Regression analysis was then performed using this subset of βNTI values and measured clinical variables to reveal factors associated with variable selection. Notably, we found that differences in gestational age at birth, measured as days postconception, were significantly associated with elevated βNTI values (*R* = 0.32, *P* < 0.0001) (Fig. 3A). Other factors, such as the influence of infection (C-reactive protein levels ([CRP]), weight, mechanical ventilation (FiO_2_), and amount of enteral nutrition, showed no such association (*P* > 0.05) (Fig. S2).

10.1128/msystems.00163-22.2 FIG S2 Mechanisms that do not underlie initial gut microbiota assembly in extremely premature infants. (A and B) Distribution of βNTI values of >2 for pairwise comparisons of samples between different patients within NICU-1 during the first week postdelivery regressed against differences in C-reactive protein levels (CRP; mg dL^−1^) (A) and weight (g) (B). (C) Fraction of inspired oxygen (FiO_2_). (D) Amount of enteral supply (mL). Regression line (blue) with confidence interval (gray) illustrates the association between βNTI values of >2 and increasing differences of referred variables. Download FIG S2, PDF file, 0.06 MB. Copyright © 2022 Seki et al. 2022 Seki et al. https://creativecommons.org/licenses/by/4.0/ This content is distributed under the terms of the Creative Commons Attribution 4.0 International license.

In order to better understand initial microbiome composition, we first tested whether observed richness was associated with gestational age at birth, but we did not observe statistically significant differences (analysis of variance [ANOVA], *P* > 0.05). Furthermore, only bacteria of the genus *Lactobacillus* were significantly less abundant in infants born after 23 weeks of gestation than infants born after 24 (ANOVA, *P* = 0.0067) and 25 weeks of gestation (ANOVA, *P* = 0.023). Next, we tested a global model for its significance and explanatory potential via RDA, including all variables listed in Table 1, yet this time only with samples obtained during the first week postdelivery (Fig. 3C). As the global model was significant (*R*^2^ = 0.65, *P* = 0.012), we proceeded to identify the most significant explanatory variable via stepwise forward selection. The amount of parenteral energy (*R*^2^ = 0.23, *P* = 0.03) was the only factor significantly associated with microbiota composition in the first week of life. Of note, postconceptional age was not associated with microbiota composition. This suggests that variable selection may be driven by unmeasured, and possibly composite, factors related to the overall developmental level of neonates that are associated with postconceptional age.

### Drift causes divergence of underlying ecological processes between neonatal wards.

**FIG 4:**
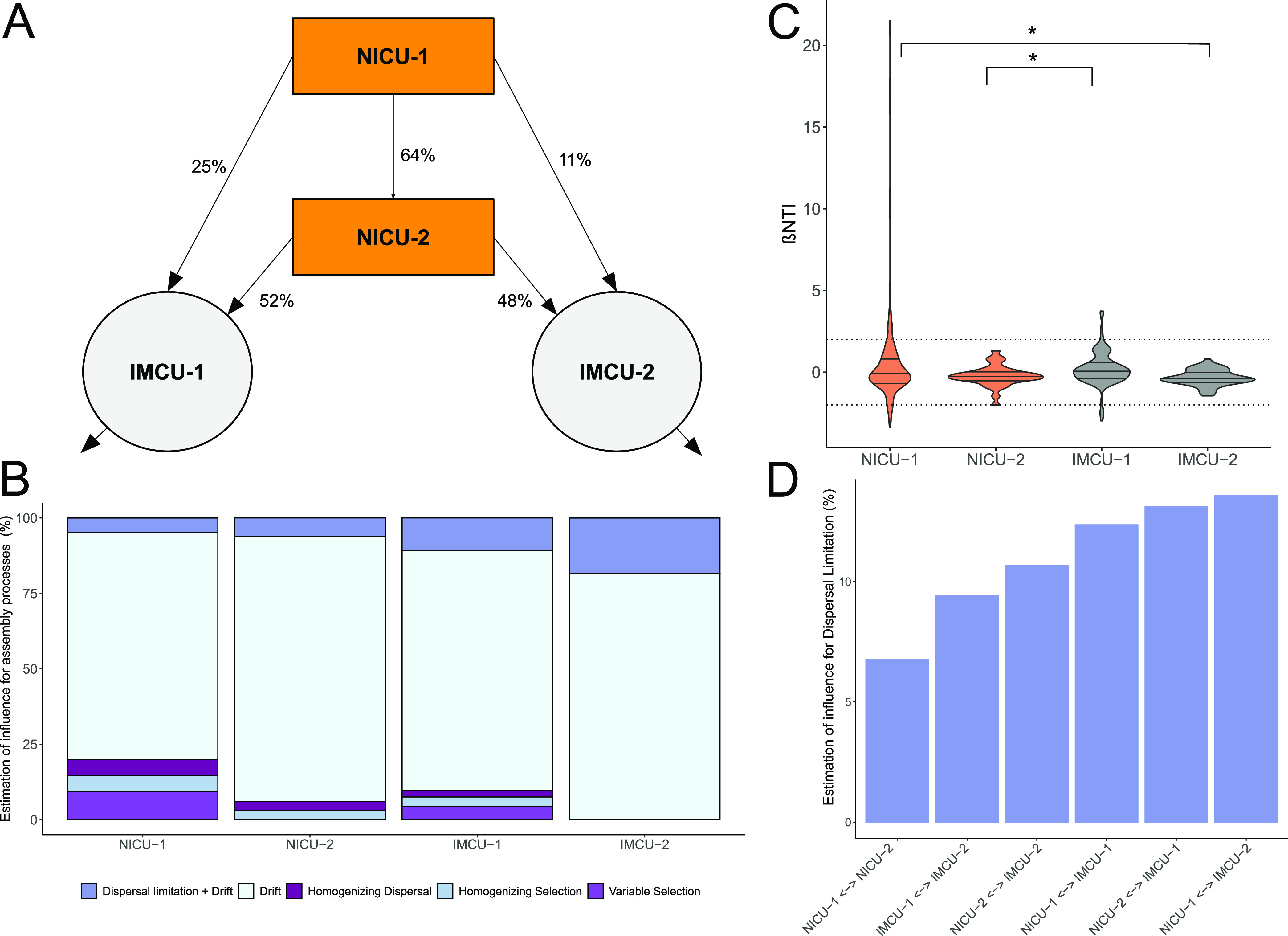
Influence of ecological processes on gut microbiota in neonatal wards. (A) Schematic illustration of transfer of premature infants between neonatal wards. (B) Contribution of individual turnover processes to observed differences in microbial community composition, resulting from pairwise comparisons of samples between different patients who were sampled during the same calendar week within the same clinical ward. (C) Distribution of βNTI values for pairwise comparisons of samples between different patients within the same clinical ward. Dashed lines indicate the range of βNTI values under the null expectation of no significant effects of selection (less than |2|). (D) Contribution of dispersal limitation to observed differences in microbial community composition, resulting from pairwise comparisons of samples between different patients who were sampled during the same calendar week between differing clinical wards. *, *P* < 0.05; ** *P* < 0.01; ***, *P* < 0.001.

Neonatal wards have been implicated as an important source of bacteria that are transferred between occupants and the room (28). Our cohort was hospitalized in four different clinical wards that are physically separated from each other and therefore can be thought of as regional metacommunities that may have different dispersal limitations due to the physical proximity of cohoused infants. However, these regional metacommunities are also connected to each other due to the directional transfer of infants between wards. All patients were initially hospitalized in neonatal intensive care unit 1 (NICU-1) and when clinically stable were then transferred to an intermediate care unit (IMCU-1 or IMCU-2). Transfers from NICU-1 to NICU-2 occurred when infants were not yet clinically stable but beds at NICU-1 were fully occupied (Fig. 4A).

The gut microbiotas of patients in IMCUs were generally more diverse (Fig. S3A). NICU-1 and NICU-2 microbiotas were different from IMCU-1 and IMCU-2 (*t* test, *P* < 0.0001), although the observed richness was similar when comparing IMCU-1 to IMCU-2 (*t* test, *P* = 0.22) and NICU-1 to NICU-2 (*t* test, *P* = 0.75). Staphylococcus was most abundant in NICU-1 (Fig. S3B), but its association with NICU-1 was not significant (ANOVA, *P* = 0.09). In general, the room was not significantly associated with the observed microbial composition (PERMANOVA, *R*^2^ = 0.01, *P* = 0.15). Microbial composition could be better explained by patient (PERMANOVA, *R*^2^ = 0.23, *P* = 0.01), as well as the combined effects of patient and age (PERMANOVA, *R*^2^ = 0.13, *P* = 0.005). However, when controlling for patient individuality, rooms explained a minor but significant fraction of the variation (*R*^2^ = 0.04, *P* = 0.01). This suggests that the gut microbiota fingerprint is highly associated with the individual patient, with further individualization proceeding over time, with minor influence of locality. Furthermore, we find that only bacteria of the genus Escherichia*-Shigella* had a minor negative correlation with the time spent in NICU-1 (Fig. S4; *R* = −0.062, *P* < 0.001). To further determine if ward residency affected ecological assembly processes, we again employed the βNTI and RC_Bray_ framework as described above. For this, βNTI values were determined across all pairwise community comparisons between samples of the same room obtained during the same calendar week. Consistent with our previous results, we observed that drift was the dominant ecological assembly process in all hospital wards. However, there were differences between the wards, with NICU-1 having the highest occurrence of variable selection (βNTI > 2) (Fig. 4B). All infants received initial care at NICU-1, and thus, NICU-1 included the youngest infants. Interestingly, βNTI values of the two NICUs were not significantly different, but NICU-1 differed from IMCU-2, and NICU-2 differed from IMCU-1 (*t* test, *P* = 0.014 and *P* = 0.01, respectively) (Fig. 4C). βNTI values between IMCU-1 and IMCU-2 were also significant (Wilcoxon test, *P* < 0.001). Direct transfers of infants from NICU-1 to IMCU-1 occurred more frequently than from NICU-1 to IMCU-2 (21% versus 12%), which might explain the slight increase of βNTI values beyond |2| in IMCU-1 compared to IMCU-2. We next evaluated interward dispersal limitation (Fig. 4D). We find that as most transfers occurred from NICU-1 to NICU-2, these wards also seem to be the least restricted by dispersal limitation (7%). However, as the fewest transfers occurred from NICU-1 to IMCU-2, dispersal limitation is higher between those wards (14%). Accordingly, RC_Bray_ values differed significantly in a comparison between “NICU-1 ←→ NICU-2” and “NICU-1 ←→ IMCU-2” (*t* test, *P* < 0.001). Generally, however, dispersal limitation was lower between NICUs and IMCUs but higher in comparison between the different types of neonatal wards.

10.1128/msystems.00163-22.3 FIG S3 Neonatal wards and their respective premature infant gut microbiota. (A) Shannon diversity indices within different neonatal wards. (B) Contribution (%) of specific bacterial genera to the observed microbiota at respective neonatal wards. *, *P* < 0.05; **, *P* < 0.01; ***, *P* < 0.001; ns, *P* > 0.05. Download FIG S3, PDF file, 0.09 MB. Copyright © 2022 Seki et al. 2022 Seki et al. https://creativecommons.org/licenses/by/4.0/ This content is distributed under the terms of the Creative Commons Attribution 4.0 International license.

10.1128/msystems.00163-22.4 FIG S4 Correlation of premature infant core microbiota to hospitalization in NICU-1. Spearman correlation for the absolute abundances of premature infant core microbiota in neonatal intensive care unit 2 (NICU-2) and intermediate care units 1 and 2 (IMCU-1 and IMCU-2, respectively) to the time a premature infant was hospitalized in neonatal intensive care unit 1 (NICU-1). Smoothed lines result from locally estimated scatterplot smoothing (LOESS) and indicate trends of development. *, *P* < 0.05; **, *P* < 0.01; ***, *P* < 0.001. Download FIG S4, PDF file, 0.8 MB. Copyright © 2022 Seki et al. 2022 Seki et al. https://creativecommons.org/licenses/by/4.0/ This content is distributed under the terms of the Creative Commons Attribution 4.0 International license.

We conclude that the observed diversification of premature infant microbiomes is largely driven by time postdelivery rather than ward residency. Interestingly, however, there seems to be an increase in dispersal based on the flow of the infants between neonatal wards, suggesting that as infants come from the same ward, they are more likely to share microbiota in a new ward. However, the general lack of homogenizing dispersal additionally suggests that although there is ongoing dispersal, we do not capture its homogenizing effects due to rapid turnover of microbiota during early succession.

### Gut microbiome assembly in healthy term-born neonates.

**FIG 5:**
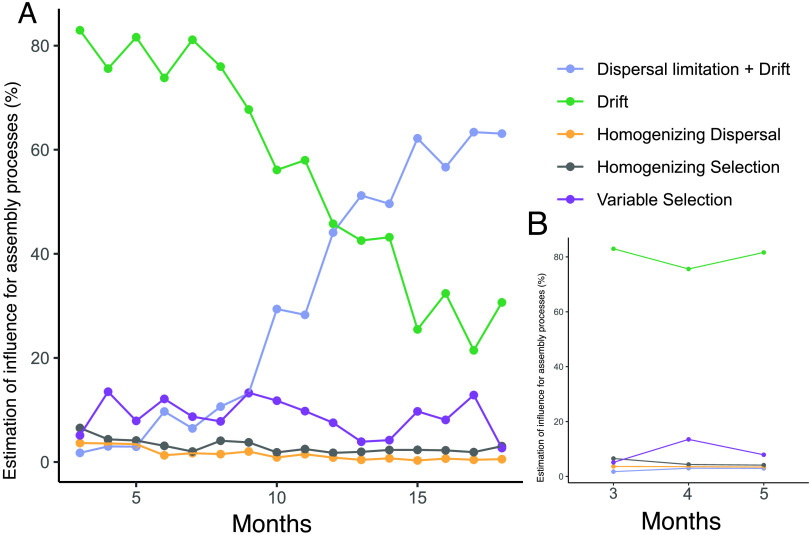
Influence of ecological processes on gut microbiota succession throughout time in healthy term-born infants. (A) Contribution of individual turnover processes to observed differences in microbial community composition resulting from pairwise comparisons of samples between different term-born infants who were sampled during the same month postdelivery. (B) Magnification of months 3 to 5.

To compare how GIT microbial community assembly progresses in extremely premature infants compared to healthy term-age infants (>38 weeks of gestation), we analyzed a publicly available data set consisting of 16S rRNA gene sequences from fecal samples of infants who were sampled over the first 18 months of life (27). It must be noted that the authors used an open-reference operational taxonomic unit approach to cluster 16S rRNA reads, while the 16S rRNA reads for the extremely premature infant cohort were clustered via clustering of amplicon sequencing variants (20). Due to this difference, as well as differences in experimental methodologies used to generate the data, direct comparisons between the two data sets should be treated with care. Despite these methodological differences, we find that drift also dominated microbial assembly and succession in term-born infants (Fig. 5A). Interestingly, dispersal limitation in combination with drift increased over time but at a much later time point than for premature infants, becoming the dominant assembly process only after 1 year postdelivery. Variable selection was most prominent early postdelivery (Fig. 5B) and remained minor but present later on, varying between 3% and 14%.

## DISCUSSION

Our study addresses the ecological processes underlying the assembly of microbes in the GIT of extremely premature infants. Initially, the gut microbiota had very low diversity. With time, the phylogenetic and functional complexity of the microbiota increased, as demonstrated by the late arrival of strictly anaerobic bacteria (Fig. 1). Our analysis suggests that this progression seems mainly driven by neutral ecological processes (Fig. 2). Furthermore, the stability of the microbiota increased with time, as marked by less frequent recursion to founder communities and increased dispersal limitation between developed and founder communities.

Stochastic processes have also been found to dominate microbial colonization during succession in other environments, such as during the formation of hypersaline microbial mats, which were initially stochastic but showed a trend toward deterministic selection in later stages of succession (4). We did not observe the emergence of such a trend for either preterm or term infants (Fig. 2B and Fig. 5). However, the gut microbiota of adults has been shown to be much more influenced by deterministic processes (8), suggesting that there may be a transition point later in childhood or puberty in which deterministic processes become more influential. Deterministic processes may underly, for example, the arrival of other functional guilds of lower-trophic chains in the developing gut (e.g., H_2_-consuming, sulfate-reducing bacteria or methanogenic archaea).

The neonatal ward environment has been implicated as an important source of microbes for the colonization of premature infant guts (28). Although we observed associations between certain microbial taxa and NICU and IMCU environments (Fig. S3 in the supplemental material), this was confounded by differences in patient age and may also be driven in part by ward-specific clinical practices such as different antibiotic regimes. It has been shown, for example, that bifidobacteria are especially susceptible to antibiotic interventions (29), whereas many potentially pathogenic *Enterobacteriaceae* are resistant to a variety of antibiotics (30). Furthermore, it was recently shown that probiotically administered bifidobacteria are less abundant in infants with very low gestational age (31). It remains unclear whether the prematurity of the host or NICU conditions suppress bifidobacterial colonization. Many clinics continue to provide bifidobacteria-containing probiotics, as they have been shown to suppress overall sepsis rates (32, 33). However, we lack fundamental knowledge concerning the colonization success of probiotics in infants, as well as a conceptual understanding to predict colonization efficacy. Our results provide a framework to guide future research into therapeutic pre- and probiotic interventions as well as ward hygiene measures. Interestingly, we observed that IMCU-1 was ecologically different from IMCU-2 (Fig. 4B and C). The design of both wards differs, with IMCU-1 having two to three bedrooms and IMCU-2 being an open ward, which potentially contributes to the observed differences. However, they both have similar clinical practices, and the average age of hospitalized infants was similar in both (IMCU-1, 61 days; IMCU-2, 55 days), in contrast to infants in NICU-1 (15 days) and NICU-2 (35 days). We partially explain the observed ecological differences based on the differential flow of infants between wards. IMCU-1, in contrast to IMCU-2, had a considerable influx of infants directly from NICU-1, in which variable selection was most prevalent. Correspondingly, variable selection is higher in IMCU-1 than IMCU-2, suggesting that infants not only shuttle microbiota between neonatal wards but also influence ward-level microbiota assembly processes.

During early microbiota succession, most of the observed bacteria are facultative anaerobes, with obligate anaerobes arriving only later and contributing toward increased richness and diversity (Fig. 1). This initial progression was predominantly stochastic, with minimal barriers to dispersal between infants, especially early postdelivery (Fig. 2A and B). Dispersal constitutes a major ecological process with important implications for the diversity in local microbial communities (19). Theoretically, higher rates of dispersal should result in a homogenizing increase of alpha diversity and decrease of beta diversity in local communities. However, we find no homogenizing effect despite the lack of dispersal limitations during initial assembly in premature (Fig. 2B), as well as term-born, infants (Fig. 5). We speculate that, as all GITs are uncolonized postdelivery and any dispersing microbe could theoretically colonize successfully, initial assembly is dominated by ecological drift. However, we did observe an increase in dispersal limitation in later periods of succession (Fig. 2B), especially between founder and developed communities (Fig. 2C). These late phases of assembly have increased compositional stability, as characterized by fewer recursions to founder states (Fig. 1C and D), suggesting that the established microbiota acts as a buffer against the arrival of novel microorganisms. This colonization resistance, in concert with drift, gives rise to the observed increase in interindividuality (Fig. 1E). Interestingly, this buffering function is established generally after the gastrointestinal tract becomes populated to the maximum of the realizable carrying capacity of the environment (Fig. 1A), which could partially explain the late increase of dispersal limitation. However, changes in diet (e.g., removal of probiotic supplementation at a corrected age of 34 weeks of gestation) also occur in this time period, so external factors may also be involved in the establishment of colonization resistance. Similarly, we observed such trends in term-born infants as well, whereby dispersal limitation increased rapidly 6 months postdelivery. Interestingly, this time frame corresponds to the typical period of introduction of novel foods besides breast milk.

Although stochastic processes appear to be the major determinant of microbiota assembly, we found that variable selection played a role shortly postdelivery, and differences in gestational age at birth partially drove initial assembly (Fig. 4A). This was reflected in part by differential abundances of *Bifidobacterium*, Escherichia-*Shigella*, Staphylococcus, and Klebsiella on day 3 (Fig. 3B). Colonization with Klebsiella spp. especially has critical implications for health, and we recently showed that Klebsiella dominance underlies the formation of a potentially pathological community state, which associates with perinatal brain injury and may thereby be involved in long-lasting morbidities (20).

We conclude that gut microbiota assembly and succession in extremely premature, as well as term-born, neonates are predominantly stochastic, with increasing dispersal limitation over time. These findings reveal ecological aspects of early-life microbiome assembly in human GITs and underline the individuality of microbiota development and the need for personalized microbiome-targeted therapeutic options to ensure proper development and maturation in premature infants.

## MATERIALS AND METHODS

### Experimental model and subject details.

Sixty extremely premature infants were enrolled between September 2017 and June 2019 at the General Hospital of Vienna as part of the PreMiBraIn study, which was approved by the ethics commission of the Medical University of Vienna (ethics number 1348/2017). Inclusion criteria were birth before the 28th week of gestation with less than 1,000 g birth weight. Infants with congenital malformations, chromosomal aberrations, maternally transmitted infectious diseases (e.g., HIV, hepatitis A/B/C, etc.), and inborn errors of metabolism were excluded. Clinical parameters of each patient were prospectively monitored by the clinical staff during hospitalization and recorded within the hospitals’ electronic database, ICIP (Philips Healthcare Systems). To minimize study bias, the general in-house standard procedures for neonatal care were followed consistently over the whole study period, including antibiotic regimen, probiotic administration, and feeding regimen. Every infant received, starting from the first day of life, the probiotic preparation Infloran (Bifidobacterium bifidum and Lactobacillus acidophilus) until the corrected age of 34 weeks of gestation. The enteral feeding regimen includes the infant’s own mother’s milk or pasteurized human donor milk. When 100 mL/kg enteral feeding was achieved, the milk was fortified with 4% bovine milk fortifier until term-equivalent age. Over the course of hospitalization, stool samples were collected 3, 7, and 14 days postdelivery and biweekly thereafter until discharge. In total, we collected 547 stool samples; each was sampled from diapers via collection tubes during patient care routines and immediately stored at −80°C until further analysis.

### Data acquisition and analysis.

We generated 16S rRNA data for term-born infants as previously described (27). We generated 16S rRNA data for extremely premature infants and corrected them for absolute abundance via qPCR (20). Both studies employed the same forward (F515, GTG YCA GCM GCC GCG GTA A-3′ [34]) and reverse (R806; GGA CTA CNV GGG TWT CTA AT-3′ [35]) primers for PCR amplification. For the premature infant data set, 16S rRNA gene counts were normalized via rarefying to even minimal sampling depth (4,000 reads per sample). Subsequently, a filter was applied to exclude ASVs that occurred in less than three samples overall (20). For the term-born infant data set, the data were rarefied at 18,429 sequences per sample (27).

Basic statistical analysis and data visualization (e.g., Student's *t* test, ANOVA, PERMANOVA, Shapiro-Wilk test, and Wilcoxon test) were performed in R version 4.0 (36) and the R packages rstatix version 0.7.0 (37), ampvis version 2.0 (38), and ggplot2 version 3.3.3 (39). The Shapiro-Wilk test was performed to test for normality of the data, and the result of this determined whether *t* tests or Wilcoxon tests were used. All *P* values were adjusted using Bonferroni’s method for unpaired *t* test and Wilcoxon rank-sum test, as well as Tukey’s *post hoc* test for ANOVA. Alpha and beta diversity indices were calculated using the R package vegan version 2.5 (40).

We employed a null model analysis to compare beta mean nearest-taxon index (βNTI) for pairwise comparisons between specific samples to assess whether their phylogenetic similarity is significantly higher or lower than expected by chance (26). The picante package was used to calculate βNTI (41). Briefly, a pairwise comparison of βNTI values indicates that two communities are phylogenetically less similar (>2) or more similar (less than −2) than by random chance and are therefore likely driven by either variable or homogenizing selection. Values greater than |2| are characteristic results of either similar or divergent selective forces that deterministically structure microbiomes. Values smaller than |2| identify stochastic processes as the main forces driving microbial community composition. To furthermore analyze ASV turnover, we used the modification of Raup-Crick (RC_Bray_) dissimilarity (22) to additionally characterize βNTI values between |2|. A significant deviation of RC_Bray_ beyond |0.95| compared to a null model indicates that given communities are assembled by either homogenizing dispersal (RC_Bray_ less than −0.95) or the combination of dispersal limitation and randomly various birth and death rates (drift) (RC_Bray_ > 0.95).
