# Supplementary figures and images for: Individuality of the Extremely Premature Infant Gut Microbiota Is Driven by Ecological Drift
Source: mSystems. 2022 Apr 27;7(3):e00163-22. doi: 10.1128/msystems.00163-22 (PMC9238403; doi:10.1128/msystems.00163-22)

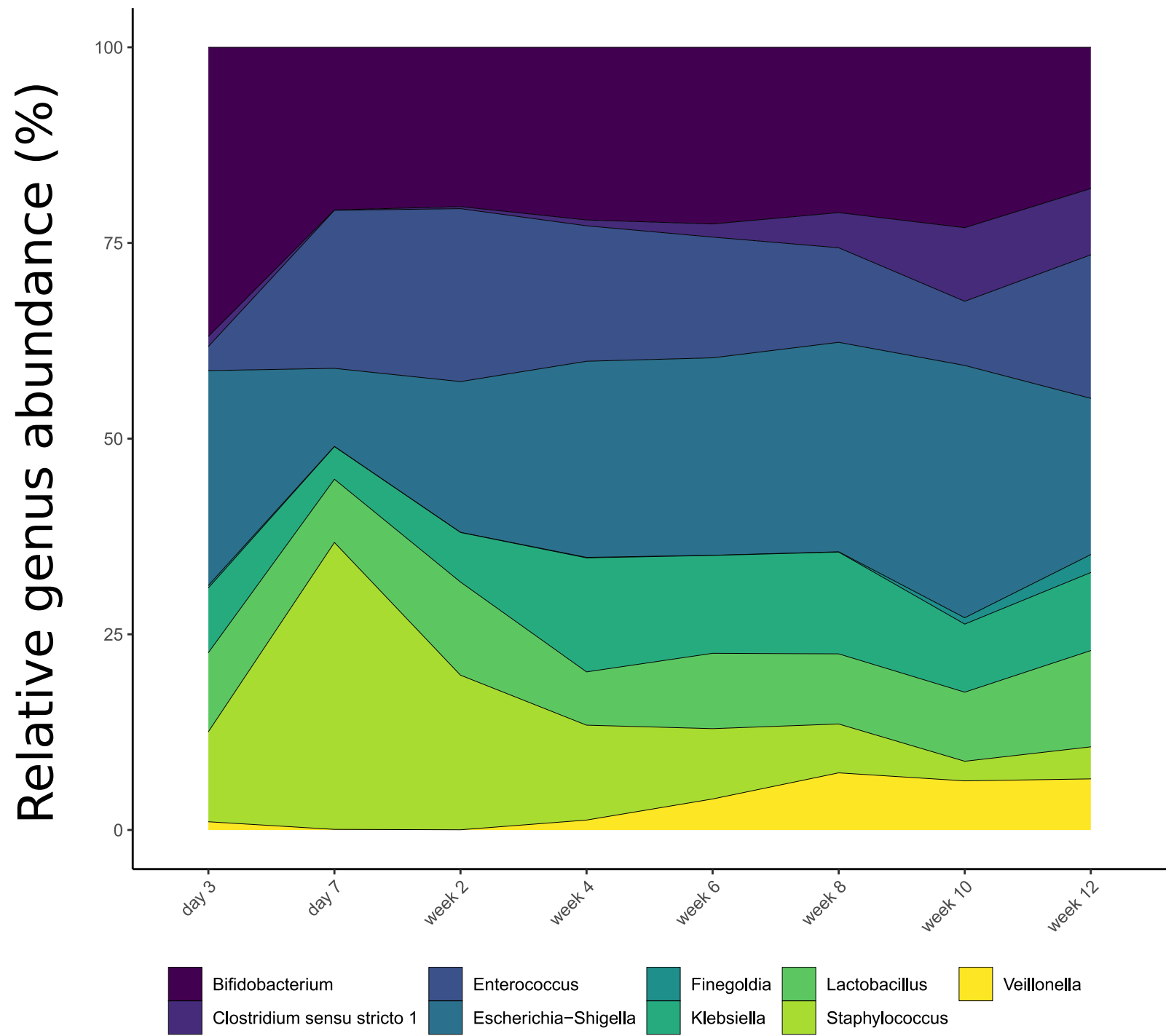

Supplement: FIG S1 [file msystems.00163-22-sf001.pdf]

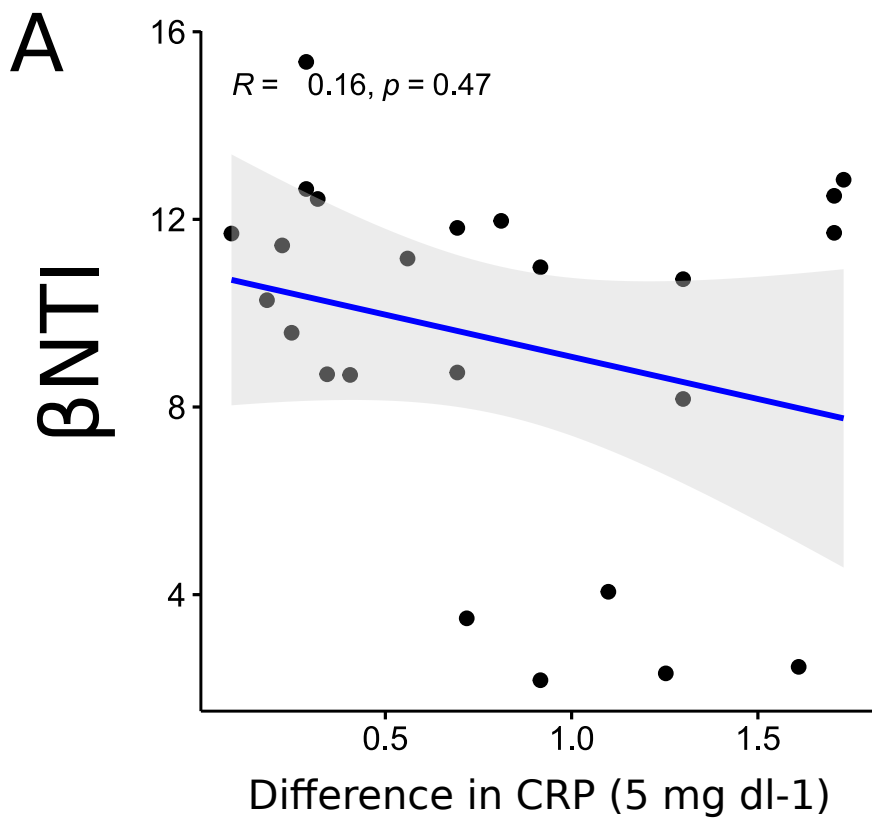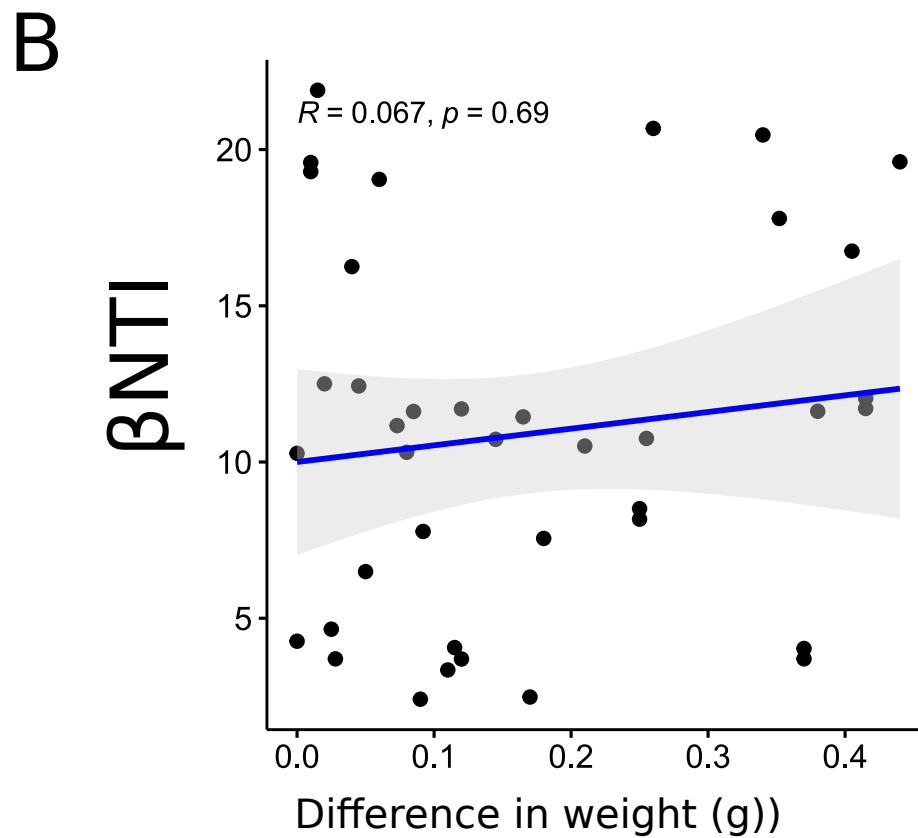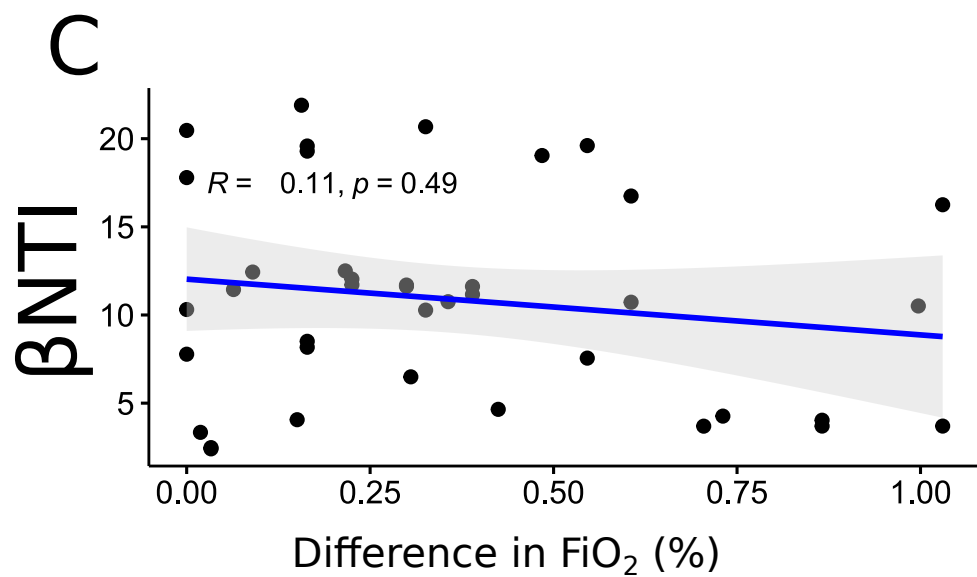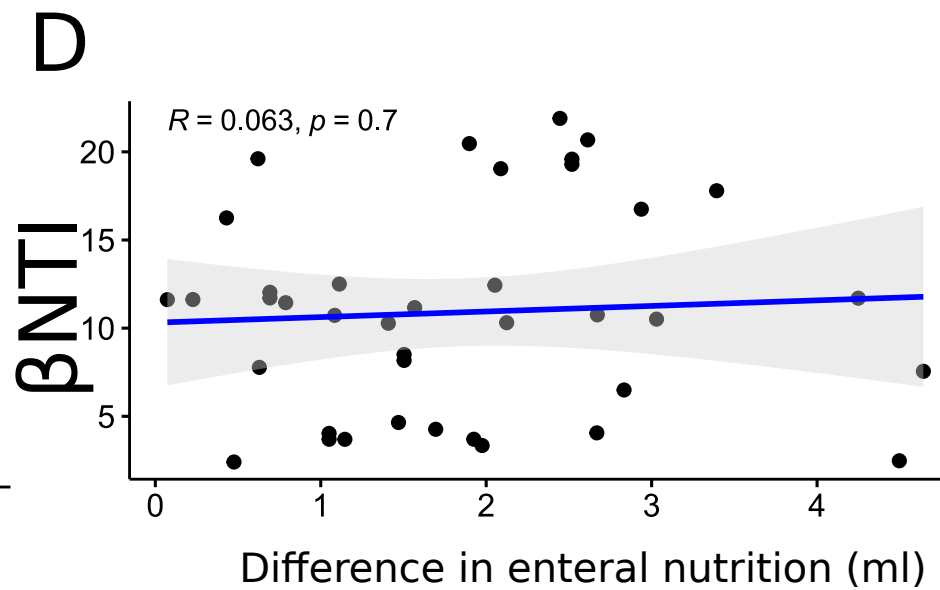

Supplement: FIG S2 [file msystems.00163-22-sf002.pdf]

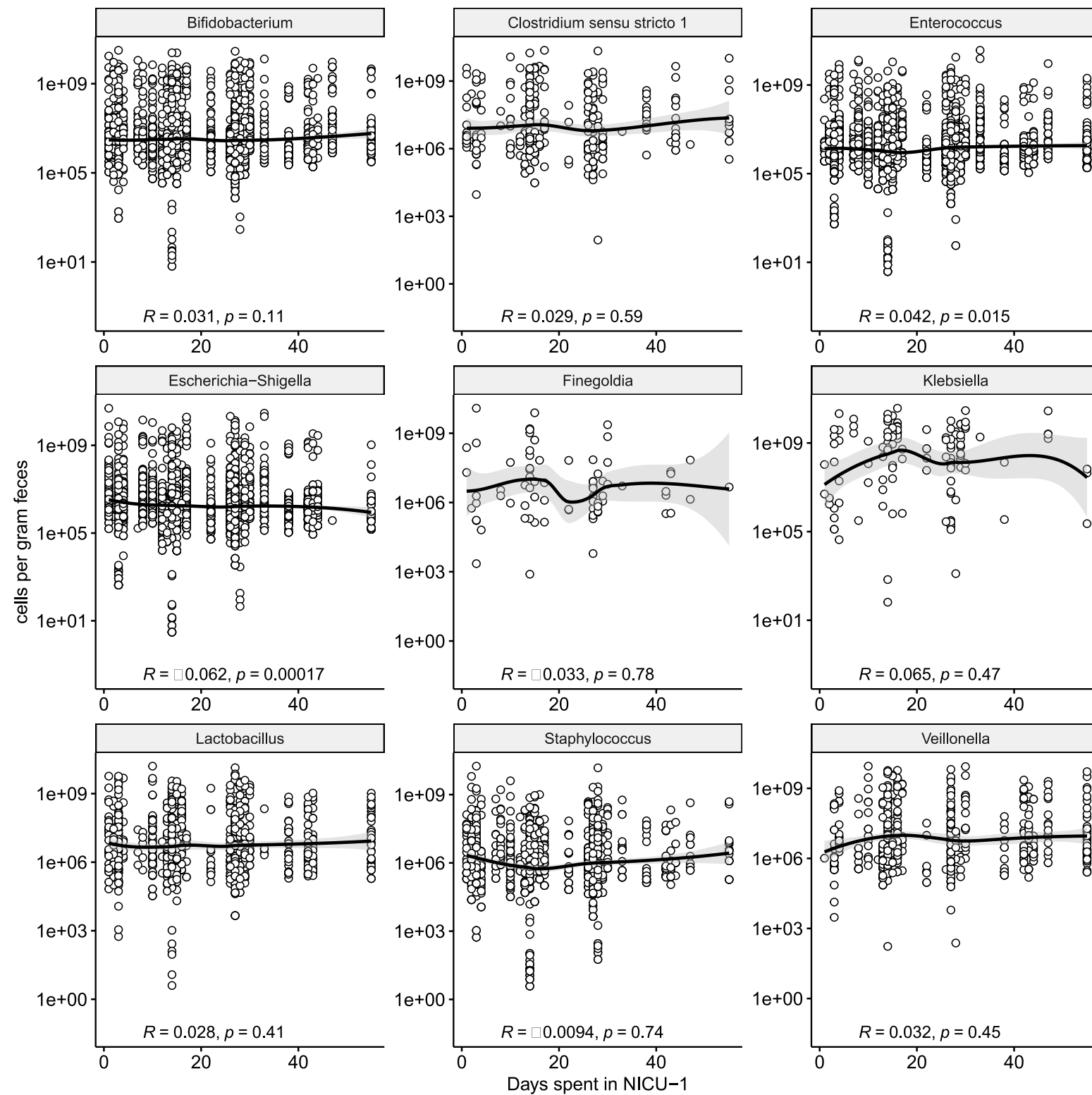

Supplement: FIG S4 [file msystems.00163-22-sf004.pdf]
