# Supplementary material for: Individuality of the Extremely Premature Infant Gut Microbiota Is Driven by Ecological Drift
Source: mSystems. 2022 Apr 27;7(3):e00163-22. doi: 10.1128/msystems.00163-22 (PMC9238403; doi:10.1128/msystems.00163-22)
Supplement: FIG S3 [file msystems.00163-22-sf003.pdf]

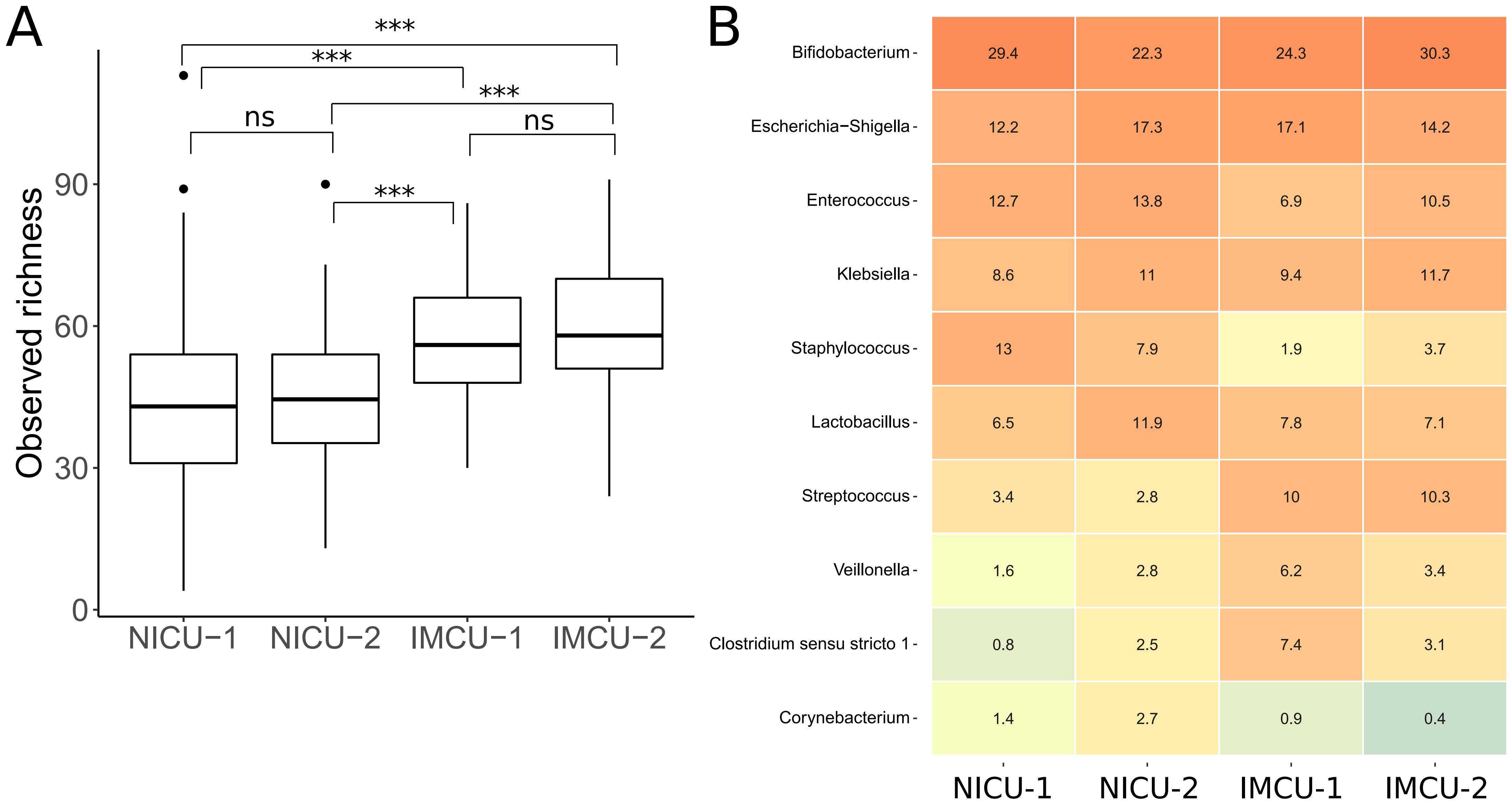

A
***
***
***
ns
ns
***
Observed richness
90
60
30
0
NICU−1
NICU−2
IMCU−1
IMCU−2
B
Bifidobacterium
29.4
22.3
24.3
30.3
Escherichia−Shigella
12.2
17.3
17.1
14.2
Enterococcus
12.7
13.8
6.9
10.5
Klebsiella
8.6
11
9.4
11.7
Staphylococcus
13
7.9
1.9
3.7
Lactobacillus
6.5
11.9
7.8
7.1
Streptococcus
3.4
2.8
10
10.3
Veillonella
1.6
2.8
6.2
3.4
Clostridium sensu stricto 1
0.8
2.5
7.4
3.1
Corynebacterium
1.4
2.7
0.9
0.4
NICU-1
NICU-2
IMCU-1
IMCU-2
